# Exploring the Antibacterial Activity of *Pestalotiopsis* spp. under Different Culture Conditions and Their Chemical Diversity Using LC–ESI–Q–TOF–MS

**DOI:** 10.3390/jof6030140

**Published:** 2020-08-19

**Authors:** Madelaine M. Aguilar-Pérez, Daniel Torres-Mendoza, Roger Vásquez, Nivia Rios, Luis Cubilla-Rios

**Affiliations:** 1 Laboratory of Tropical Bioorganic Chemistry, Faculty of Natural, Exact Sciences and Technology, University of Panama, Panama 0824, Panama; mad25aguilar@gmail.com (M.M.A.-P.); dtorresm.507@gmail.com (D.T.-M.); royi071123@gmail.com (R.V.); 2 Vicerrectoría de Investigación y Postgrado, University of Panama, Panama 0824, Panama; 3 Department of Microbiology, Faculty of Natural, Exact Sciences and Technology, University of Panama, Panama 0824, Panama; toxogondii@gmail.com

**Keywords:** Endophytic fungi, *Hyptis dilatata*, *Pestalotiopsis mangiferae*, *Pestalotiopsis microspora*, chemical elicitors, antibacterial activity, LC–ESI–Q–TOF–MS

## Abstract

As a result of the capability of fungi to respond to culture conditions, we aimed to explore and compare the antibacterial activity and chemical diversity of two endophytic fungi isolated from *Hyptis dilatata* and cultured under different conditions by the addition of chemical elicitors, changes in the pH, and different incubation temperatures. Seventeen extracts were obtained from both *Pestalotiopsis mangiferae* (*man-1* to *man-17*) and *Pestalotiopsis microspora* (*mic-1* to *mic-17*) and were tested against a panel of pathogenic bacteria. Seven extracts from *P. mangiferae* and four extracts from *P. microspora* showed antibacterial activity; while some of these extracts displayed a high-level of selectivity and a broad-spectrum of activity, *Pseudomonas aeruginosa* was the most inhibited microorganism and was selected to determine the minimal inhibitory concentration (MIC). The MIC was determined for extracts *man-6* (0.11 μg/mL) and *mic-9* (0.56 μg/mL). Three active extracts obtained from *P. mangiferae* were analyzed by Liquid Chromatography-Electrospray Ionization-Quadrupole-Time of Flight-Mass Spectrometry (LC–ESI–Q–TOF–MS) to explore the chemical diversity and the variations in the composition. This allows us to propose structures for some of the determined molecular formulas, including the previously reported mangiferaelactone (**1**), an antibacterial compound.

## 1. Introduction

The World Health Organization (WHO; Geneva, Switzerland) has established an urgent pathogen list of antibiotic-resistant bacteria to guide the research, discovery, and development of new antibiotics. This list includes carbapenem-resistant *Pseudomonas aeruginosa* and *Enterobacteriaceae* and third generation cephalosporin-resistant bacteria as critical priorities as a result of the continuous and indiscriminate use of antibiotics, not only in the treatment of human diseases, but also in animals [1,2]. This list includes antifungal compounds [3].

Pharmaceutical conglomerates have abandoned this field of research due to the high costs. Despite the efforts made in recent years, e.g., investment in research and development (R&D) as well as in scientific and technological research, the strategies for the search of new antibiotics and antifungals remain uncertain [4,5]. In this context, natural products produced by endophytic fungi provide an alternative to supply new molecules with antimicrobial activities [6,7,8,9].

Endophytic fungi spend a large part of their life cycle inside the tissue of the host organism without causing apparent damage [10]. In the last 15 years, interest in endophytic fungi has grown exponentially because of their ability to produce a wide range of secondary metabolites with diverse and important biological activities. Plant endophytes are considered one of the least studied groups of microorganisms and have proven to be a source of natural products and therefore provide a way to discover novel compounds with biological activities [11,12]. Two species of the genus *Pestalotiopsis*, isolated from *Hyptis dilatata* (Labiatae), a plant that is distributed in the north and east of the Republic of Panama and that is known for producing abietane and pimarane diterpenes [13] were selected for these studies.

The genus *Pestalotiopsis* is considered a vast source of natural products from which more than 300 compounds have been isolated and characterized, including terpenoids, polyketides, chromones, quinones, coumarins, lactones, and nitrogen-containing molecules with a wide range of biological activities such as antifungal, antibacterial, anticancer, antioxidant, antiparasitic, antihypertensive, anti-inflammatory, and neuroprotective activities [14,15,16,17,18,19]. Previously, our group reported the isolation of a set of eleven compounds (see Figure S1) from the crude extract of *P. mangiferae*, including a polyhydroxylated macrolide named mangiferaelactone [20]; the crude extract showed growth inhibition against *Listeria monocytogenes* (29 mm diameter inhibition zone), and showed a minimal inhibitory concentration (MIC) of 1.69 mg/mL and 0.55 mg/mL against *L. monocytogenes* and *Bacillus cereus*, respectively. This compound belongs to the nonalide class that is associated with important biological activities such anticancer, antifungal, antibacterial, and antiviral activities. Its synthesis has been developed by different research groups [21,22,23]. Compounds such as taxol, isopestacin, and some polyketides have been isolated from *P. microspora* [24,25,26,27].

The production of secondary metabolites by microorganisms could be impacted by environmental dynamics, such as growing conditions, which include biotic and abiotic factors [28]. Therefore, the selective variation of these parameters during the cultivation of fungi [29,30] and/or the induction of stress through competition with other microorganisms in a co-culture represent interesting ways to generate greater activity, chemical diversity, and novel active molecules [31,32,33]. Hence, the opportunity to modify culture conditions allows for the optimization of secondary metabolite production [34]. Therefore, given the growing interest in enhancing the production of secondary metabolites by endophytic fungi, the study of the methods and strategies to stimulate the gene clusters responsible for the biosynthesis of new molecules has been intensified and could include chemical or physical factors [35,36]. For example, the use of metallic ions, organic and inorganic compounds, pH, and incubation temperature to optimize the production of enzymes or secondary metabolites have been described [37,38,39,40,41,42,43,44,45,46,47].

For this study, we focused on modifying the conditions of the culture medium by varying abiotic parameters and through this, activate fungal silent gene clusters [48,49,50] in *P. mangiferae* Hd08 and *P. microspora* Hd18 in order to increase the chemical diversity and to detect new antibacterial activities. 

## 2. Materials and Methods 

### 2.1. Chemicals and Reagents

All of the following chemicals were acquired from Sigma–Aldrich^®^ (Sigma–Aldrich, St. Louis, MO, USA): arginine, glutamic acid, CuSO_4_, CaCl_2_, FeSO_4_, tri-sodium citrate dihydrate, dimethyl sulfoxide (DMSO), and formic acid (FA). Ethyl acetate, acetone, and methanol used for extraction were American Chemical Society grade (Tedia^®^, Tedia Company Inc., Fairfield, OH, USA). The methanol for the liquid chromatography-mass spectrometry (LC–MS) analysis was LC–MS grade (J. T. Baker^®^, Avantor Performance Materials, Inc., Center Valley, PA, USA).

### 2.2. Isolation and Identification of Fungal Isolates

A healthy specimen of *Hyptis dilatata* (Labiatae) was collected in La Mesa, Veraguas Province in the Republic of Panama, in November 2010. An exsiccate from the plant material was deposited in the Herbarium of the University of Panama (PMA 084861). Mature leaves were surface sterilized as we previously reported in [51], and small fragments were cultured on 2% malt-extract agar (MEA; Difco^TM^, Becton, Dickinson and Co., Sparks, MD, USA) under sterile conditions. Strains Hd08 and Hd18 were further isolated from the collection plate and successively re-plated until pure strains were obtained. Pure fungal strains were stored at −80 °C in a cryoprotectant solution of 10% glycerol and were preserved in the collection of the International Cooperative Biodiversity Group (ICBG) at the University of Panama. The identification of endophytic fungi was carried out as described previously [20]. Briefly, the total genomic DNA of each strain was isolated from fresh mycelium following U’Ren et al. [52]. Polymerase chain reaction (PCR) was used to amplify the nuclear ribosomal internal transcribed spacers and 5.8s gene (ITS rDNA; ca. 600 bp), and the first ca. 600 bp was sequenced bidirectionally [52]. The entire sequences for each strain were compared to the nucleotide database of the National Center for Biotechnology Information (NCBI) using the Basic Local Alignment Search Tools (BLAST^®^) Website. 

### 2.3. Media Preparation and Cultivation of Fungal Strains

Strains Hd08 and Hd18 were reactivated aseptically on Petri dishes containing potato dextrose agar (PDA; Difco^TM^, Becton, Dickinson and Co., Sparks, MD, USA) and incubated at 26 °C for seven days. Then, the mycelium was removed using a sterile spatula and was placed in sterile water to obtain a homogeneous solution. This solution was poured on the surface of Petri dishes (145 × 90 mm) containing MEA for all of the experimental conditions. After 15 days of incubation, the material was extracted, and the amount of crude organic extract was measured. Sterile controls were established for all of the experiments.

Chemicals as elicitors. MEA was prepared as indicated on the label; the resulting pH was measured using a pH meter (Thomas Scientific, Swedesboro, NJ, USA). The medium was then sterilized at 121 °C. When it had cooled to 45 °C, each of the elicitors was added, mixed well, and poured on Petri dishes (145 × 90 mm), and the strains then were inoculated and incubated at 26 °C for 15 days.

pH as an elicitor. MEA was prepared as described above and buffered with a 50 mmol/L tri-sodium citrate dihydrate solution to set values of 4.0, 4.6, and 5.6. The chemical elicitors were CaCl_2_ and CuSO_4_. The strains then were inoculated, and the plates were incubated at 26 °C for 15 days.

Incubation temperature as an elicitor. MEA was prepared and buffered at pH = 4.0 by adding CaCl_2_ or CuSO_4_, as described above. The incubation temperatures were set at 24, 28, and 30 ± 2 °C in the incubation chamber (Sheldon Manufacturing, Inc., Cornelius, OR, USA) for 15 days.

### 2.4. Extraction and Sample Preparation

In all of the experiments, after the incubation time, the mycelium was cut into small pieces and placed into a 1 L beaker and 500 mL of ethyl acetate was added. After 30 min, the mixture was triturated and homogenized using a Polytron^®^ (Brinkmann Instruments, Westbury, NJ, USA) and subsequently filtered through filter paper (Whatman No. 7) using a vacuum. The organic solvent was evaporated under a vacuum at 30 °C using a rotary evaporator. The resulting crude extract was re-dissolved in acetone and transferred to scintillation vials, which were previously labeled and weighed, and then evaporated on a Speed Vac^®^ Plus (Thermo Savant^TM^, Thermo Fisher Scientific, Waltham, MA, USA) for 24 h. Thereafter, the amount of extract was determined.

### 2.5. Antibacterial Activity

Tested microorganisms: Among the microorganisms used for the antimicrobial test, eight strains (*Bacillus cereus* CECT 5050, *Escherichia coli* CECT 433, *Kocuria rhizophila* CECT 241, *Legionella pneumophila* CECT 7109, *Listeria monocytogenes* CECT 935, *Pasteurella multocida* CECT 962, *Salmonella enterica* CECT 7161, *Salmonela enterica* CECT 7160, and *Shigella flexneri* CECT 4804) were acquired from the Spanish Types Culture Collection of the University of Valencia, Spain, and eight strains (*Enterobacter cloacae* ATCC 13047, *Enterococcus faecalis* ATCC 19433, *Klebsiella pneumonia* ATCC 13883, *Klebsiella pneumonia ozaenae* ATCC 11296, *Proteus vulgaris* ATCC 9484, *Pseudomonas aeruginosa* ATCC 10145, *Staphylococcus aureus* ATCC 25923, and *Streptococcus oralis* ATCC 35037) were acquired from the American Type Culture Collection.

In vitro bacterial growth inhibition: The antibacterial activity of each extract was determined through the susceptibility test of the British Society for Antimicrobial Chemotherapy (BSAC) [53]. The turbidity standard (0.5 McFarland Turbidity Standard) was a BaCl_2_ solution which absorbance (0.08–0.10 at 625 nm) was verified in a spectrophotometer (Spectronic 21, Bausch & Lomb, Rochester, NY, USA). The solution was stored in the dark at 24 ± 2 °C. The bacterial inoculum of the seventeen pathogenic strains were prepared in Trypticase-Soy Agar (TSA; Bacto^TM^, Becton, Dickinson and Co., Sparks, MD, USA) for 24 h. Thereafter, five colonies were picked up and transferred into a tube containing a saline and isotonic solution, then visually compared to the turbidity standardas previously reported [54]. 

Evaluation of the minimal inhibitory concentration (MIC): A broth dilution susceptibility testing method was applied for the determination of the (MIC) [55], using a stock solution prepared by adding 15 mg of the organic extract in 3 mL of Trypticase-Soy Broth (TSB). Serial dilutions of the organic extract (3.33 μg/mL, 1.67 μg/mL, 0.56 μg/mL, 0.18 μg/mL, 0.061 μg/mL, 0.0021 μg/mL, and 0.007 μg/mL) and positive control (gentamycin sulfate: 104.5 μg/mL, 35.0 μg/mL, 11.6 μg/mL, and 3.87 μg/mL) were performed. Each solution was inoculated with 50 μL (0.5 McFarland) of a culture of *Pseudomonas aeruginosa* and incubated at 37 °C for 18 h. The negative control was DMSO. A sterile culture media control was also used. Each assay was performed in duplicate.

### 2.6. Analysis of Organic Extracts by LC–MS

LC–MS analysis was carried out in an Agilent 1290 Infinity LC System (Agilent Technologies, Santa Clara, CA, USA) using a Zorbax^®^ Eclipse Plus (1.8 μm) C_18_ reverse phase LC column, 100 × 3 mm (Agilent Technologies, Santa Clara, CA, USA). The mass spectrometer was a micrOTOF-QIII (Bruker Daltonics, Billerica, MA, USA) supplied with an electrospray ionization (ESI) source. For the positive mode Electrospray Ionization-Quadrupole-Time of Flight-Mass Spectrometry (ESI+–Q–TOF–MS) analysis, extracts were re-dissolved in methanol and filtered through a 0.45 μm cellulose acetate membrane filter. Solutions of 0.5 μg/mL were prepared, and aliquots of 10 μL were injected. The chromatographic analysis was carried out using a 37 min step gradient (UHPLC) run using mixtures of methanol and acidified water (99.9% H_2_O-0.1% FA) as mobile phase, starting from: (a) 5–95% MeOH-H_2_O for 2 min; (b) a 25 min gradient from 5:95 methanol:H_2_O to 100% methanol; (c) 100% methanol for 8 min. The column was returned to the initial condition for 2 min. Prior to collecting the data, two level of calibration were employed; before the analysis, an external calibration was performed using an Agilent ESI-L Low-Calibration Tuning Mix, and during the evaluation of each sample, we used hexakis (1H, 1H, 2H-difluoroethoxy)-phosphazene (*m/z* 622.0290 [M + H]^+^; Synquest Laboratories, Alachua, FL, USA) as an internal reference, for the lock mass calibration.

## 3. Results

### 3.1. Culture Conditions

The results obtained from evaluating the changes in culture conditions are listed in Table S1. The addition of chemical elicitors impacted the amount of crude extract obtained. In *P. mangiferae*, the best result was achieved in presence of Fe^2+^ and Ca^2+^ ion (*man-3*, 256.0 mg and *man-4*, 232.0 mg); in *P. microspora*, under the presence of Cu^2+^ and Ca^2+^ ions (*mic-4*, 297.0 mg and *mic-5*, 329.0 mg).

Low pH values increased the amount of crude extract obtained. For both species, the best results were obtained at pH = 4.0 and Cu^2+^ as elicitor: in *P. mangiferae* (*man-9*, 264.3 mg); in *P. microspora* (*mic-9*, 260.0 mg).

The third factor to consider was the incubation temperature. Maintaining constant the pH at 4.0, we found that the highest amount of extract in *P. mangiferae* was at 30 °C for both elicitors (*man-14*, 528.0 mg; *man-17* 448.0 mg); the production of extract using Ca^2+^ was 34-fold higher and 1.7-fold higher using Cu^2+^ compared to the amounts obtained in phase II. For *P. microspora*, the highest amount of extract was obtained at 24 °C using Cu^2+^ as elicitor (*mic-15*, 1194.0 mg) that was 4.6-fold higher that the obtained amount in phase II.

### 3.2. Antibacterial Activity

A total of 34 extracts were assayed against a panel of pathogenic bacteria in a preliminary antibacterial test (disc diffusion method, mm), and only 11 extracts showed growth inhibition: seven from *P. mangiferae* and four from *P. microspora* (Table 1). These extracts were capable of inhibiting the growth of 13 of 17 pathogenic bacterial strains. Larger inhibition zones were observed against *P. aeruginosa* (12.5 mm) and *L. monocytogenes* (11.0 mm). Extract *man-6* displayed the highest broad-spectrum of antimicrobial activity (inhibited seven pathogenic strains), followed by extracts *man-9* and *mic-9* (both inhibited six pathogenic strains). Nevertheless, the culture condition for *man-6* (CaCl_2_, pH = 4.0 and T = 26 °C) induced one of the lowest amounts of organic extract (15.4 mg). The culture condition for extracts *man-9* and *mic-9* (CuSO_4_, pH = 4.0, T = 26 °C) were more favorable. 

Extracts *man-15* and *mic-11* exhibited selectivity against *P. aeruginosa*, the most sensitive strain. To determine the MIC using *P. aeruginosa*, five of the eleven extracts were selected. In this experiment, only extracts *man-6* (0.11 μg/mL) and *mic-9* (0.56 μg/mL) demonstrated growth inhibition.

Hence, to correlate the chemical profile with the antibacterial activity against *P. aeruginosa*, three samples were selected from *P. mangiferae* to be analyzed by LC–MS: (1) one active, *man-7*; (2) one with broad-spectrum activity, *man-9* (12.5 mm inhibition zone); and (3) one with selective activity, *man-15* (9.5 mm inhibition zone).

To our knowledge, there are only two reports of secondary metabolites from *P. mangiferae* [20,56].

### 3.3. Evaluation of the Chemical Diversity

**Figure 1:**
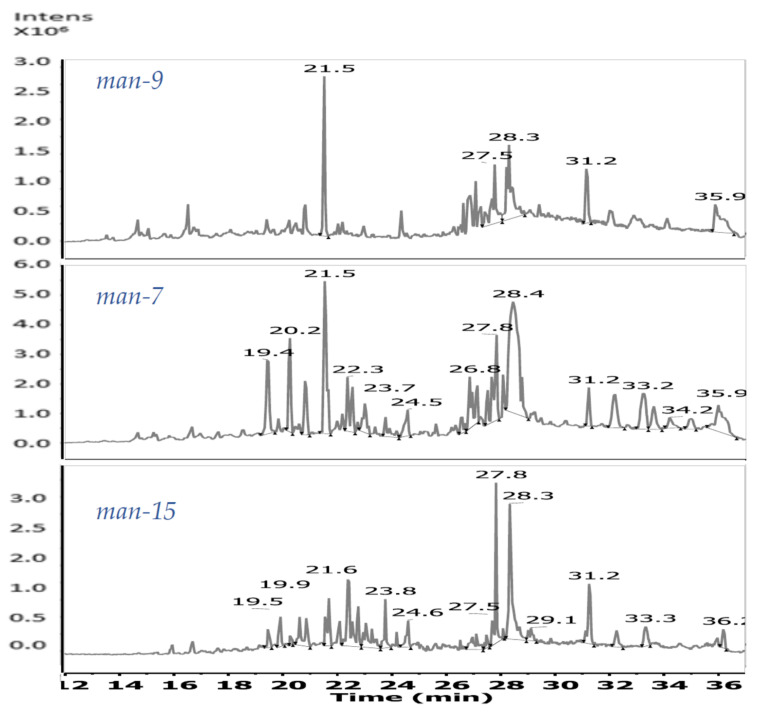
Total ion chromatograms (TICs) of extracts *man-9*, *man-7,* and *man-15*.

Table 2 lists the principal molecular ions present in the analyzed extracts and their determined molecular formulas. Extracts *man-7* and *man-15* showed a similar chemical composition; nevertheless, at least four compounds were only present in extract *man-15*. The molecular ions are linked, mainly to polyoxygenate compounds, but some nitrogenous are present too. In all three extracts (*man-7*, *man-9*, *man-15)*, the presence of mangiferaelactone was determined (retention time *t*_R_ 21.55–21.59 min; *m/z* 401.2017 [M + H]^+^); none of the other previously isolated compounds from *P. mangiferae* was detected as principal components of the analyzed samples (see Figure S1). The pseudo molecular ion *m/z* 338.341 [M + H]^+^, which appeared at *t_R_* 28.3 min, has been established as a possible molecular formula (calculated for C_22_H_44_NO). This compound appeared in the chromatograms of extracts *man-9* and *man-15*, but it was absent in the *man-7* extract (Figure 1, Table 2), suggesting that it could be responsible for the antibacterial activity against *P. aeruginosa*.

**Figure 2:**
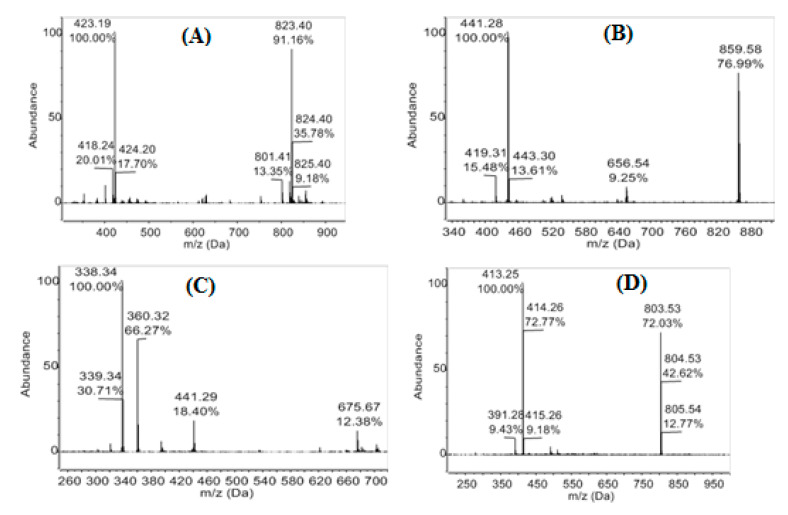
Mass spectra of selected peaks. (**A**) Mangiferaelactone at *t*_R_ 21.5 min in extracts *man-7*, *man-9*, and *man-15*. (**B**) Peak in extract *man-7* at *t*_R_ 28.4 min. (**C**) Peak in extracts *man-9* and *man-15* at *t*_R_ 28.3 min. (**D**) Peak in extracts *man-7* and *man-15* at *t*_R_ 27.8 min.

Chemical diversity of the three extracts from *P. mangiferae* (*man-7*, *man-9,* and *man-15*) was analyzed by LC–ESI–Q–TOF–MS. The total ion chromatograms for each sample are presented in Figure 1. The peak at *t*_R_ 21.5 min is common to all three analyzed samples. The MS spectrum of this peak showed ions [M + H]^+^ and [M + Na]^+^ at *m/z* 401.217 and 423.199, respectively. Additionally, two ion clusters were detected: M_2_H^+^ and M_2_Na^+^ at *m/z* 801.424 and 823.407, respectively (Figure 2A), which matched the MS data for mangiferaelactone, a previously characterized compound. This compound had a relatively lower concentration in sample *man-15*. Based on its selectivity against *P. aeruginosa* (9.5 mm inhibition zone), this compound could be proposed as the major component of the extract, for example, the peak at *t*_R_ 27.8 min with [M + H]^+^, [M + Na]^+^, and [2M + Na]^+^ ions at *m/z* 391.283, 413.261, and 803.536, respectively (Figure 2D). The polar section of *man-15* was the most complex, indicating a higher level of chemical diversity then *man-7* and *man-9*. 

Extract *man-7* showed a higher relative concentration among the components of the polar section (peaks at *t*_R_ 19.4, 20.2, 20.7, and 21.5 min). Its moderate polarity section included a peak at *t*_R_ 28.4 min with [M + H]^+^,[M + Na]^+^, and [2M + Na]^+^ ions at *m/z* 419.308, 441.276, and 859.579, respectively (Figure 2B), compared with extracts *man-9* and *man-15* that had a peak at *t*_R_ 28.3 min with [M + H]^+^, [M + Na]^+^, and [2M + H]^+^ ions at *m/z* 338.337, 360.319, and 675.670, respectively (Figure 2C).

## 4. Discussion

As we mentioned above, there are only two reports related to the isolation and characterization of secondary metabolites from *P. mangiferae*; early culturing under two different conditions produced antibacterial compounds mangiferaelactone (**1**) and 4-(2,4,7-trioxa-bicyclo[4.1.0]heptan-3-yl) phenol (see Figure S1) [20,56]. Neither one was among the main components present in the extracts analyzed here by high resolution LC–MS, nor the other compounds with a low molecular weight.

Our results showed that in this initial study, *Pestalotiopsis* showed prolific antibacterial activity. The results of Table 2 indicate that each extract had a different biological activity profile. This means that the chemical composition changes in diversity and concentration.

The changes in the culture conditions played a role in differences in the chemical diversity of *P. mangiferae* and the genus *Pestalotiopsis*. This was confirmed by the wide range of the preliminary antibacterial activities determined for each of the extracts and through the LC–HRMS analysis of the three extracts. According to the revised reviews and recent publications (Table S2) on the secondary metabolites isolated from the genus *Pestalotiopsis* and the antibacterial activity previously determined, most of the molecular formulas for the metabolites reported here did not match with those reported earlier for the genus. Nonetheless, taking into account the previously isolated compounds from the genus *Pestalotiopsis* or from *P. mangiferae* and the molecular formula obtained through high-resolution mass spectrometry, we proposed some molecular structures; also, in most of the cases they are related with a previously isolated compound with antibacterial or antifungal activity. 

Five major reviews on the chemistry and bioactivity of the genus *Pestalotiopsis* were published until 2017 [14,15,16,17,18]. An exhaustive exploration of the available compounds’ structures allowed us to establish that the majority of the compounds present in the analyzed extracts of *P. mangiferae* have not been isolated from a species of the genus *Pestalotiopsis*. Nevertheless, it could be proposed that they belong to three of the main classes of compounds isolated from the genus, namely: (a) polyketides/polyols derivatives; (b) terpenoids/triterpenoids; and (c) nitrogen-containing compounds.

**Figure 3:**
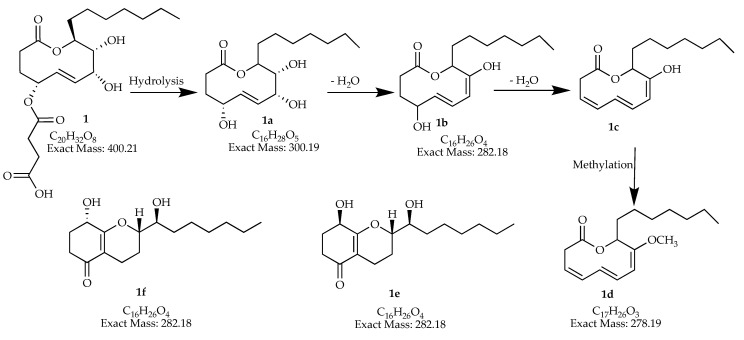
Proposed polyketide/polyol derivatives **1b**,**d** that could be present in extracts *man-7*, *man-9*, and *man-15,* having as a precursor compound **1** and the intermediates **1a**,**c**. Polyketides **1e**,**f** previously isolated from the genus *Trichoderma* with same molecular formula as **1b**.

The major group of compounds present in the analyzed extracts could belong to polyketide/polyol derivatives. For example, we proposed the hydrolysis of mangiferaelactone (**1**) (C_20_H_32_O_8_) that will result in the hypothetical trihydroxylactone (**1a**) (not-yet-detected by MS-analysis), its successive dehydration and methylation could lead to two lactones (**1b**) (C_16_H_26_O_4_) and (**1d**) (C_17_H_26_O_3_), respectively (Figure 3). Compound (**1b**) has the same molecular formula as koninginins B (**1e**; C_16_H_26_O_4_) and E (**1f**; C_16_H_26_O_4_) isolated from the genus *Trichoderma* [57,58,59], and they probably have the same precursor as compound (**1a**) (C_16_H_28_O_5_). 

**Figure 4:**
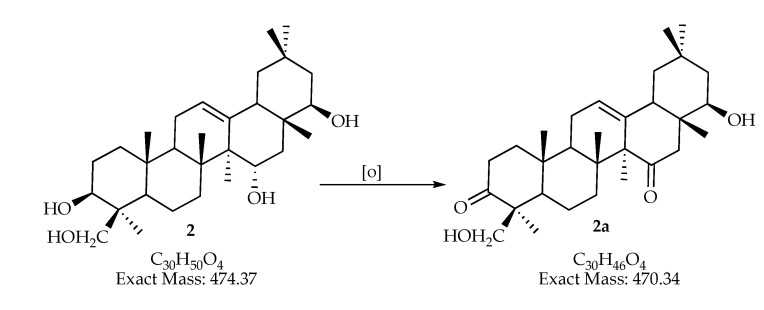

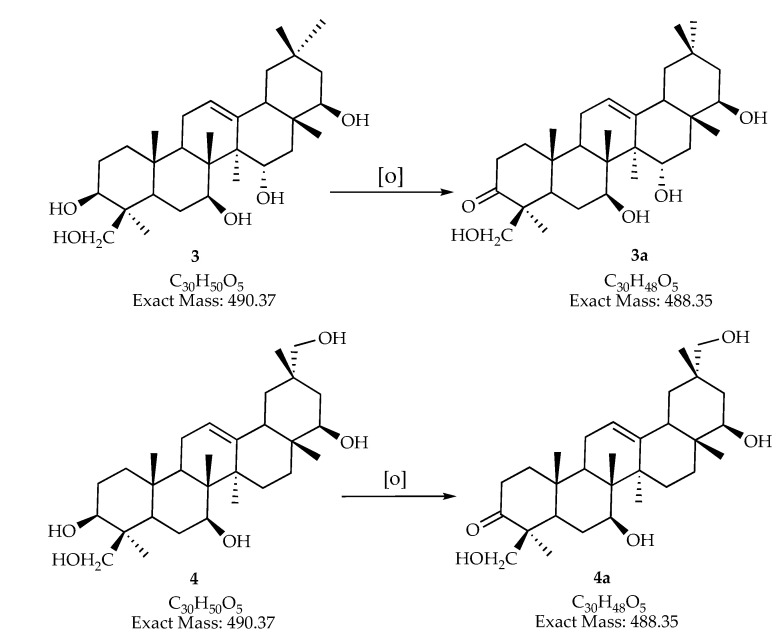
Proposed structure for compounds **2a**, **3a**, and **4a** based on the molecular formula determined by HRMS in extracts *man-7* and *man-15* and their proposed biosynthetic precursor.

Two molecular formulas could correspond to triterpenoids (C_30_H_46_O_4_/ C_30_H_48_O_5_). From the genus *Pestalotiopsis,* only oleanane- and ursane-type triterpenes have been isolated [14,17,60]. Ursane-type triterpenes have been reported when to the culture medium was added ursolic acid [61]. Nevertheless, oleanane-type were isolated (15α)-15- hydroxysoyasapogenol B (**2**), (7*β*, 15*α*)-7, 15-dihydroxysoyasapogenol B (**3**) and (7*β*)-7, 29-dihydroxysoyasapogenol B (**4**) from *Pestalotiopsis clavispora* [60], together with ursolic acid. These three compounds (**2**–**4**) could be synthesized by biological oxidation mechanism derived in one of the triterpenoids **2a**, **3a** or **4a**, respectively; they have the molecular formula C_30_H_46_O_4_ or C_30_H_48_O_5_ established by high resolution MS in extracts *man-7* and *man-15* (Figure 4).

**Figure 5:**
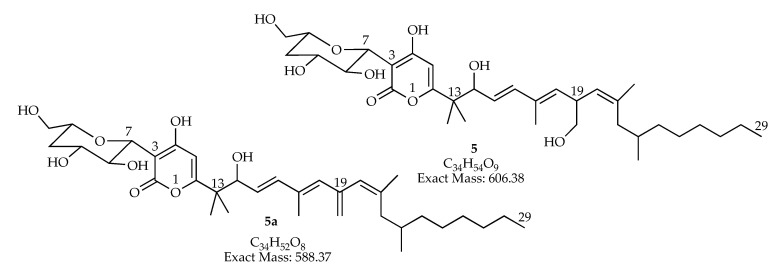
Dehydration of the polyketide derivative fusapirone **5** could produce compound **5a** with a molecular formula C_34_H_57_O_8_, determined in extracts *man-7* and *man-15.*

In our results two molecular ions have the same molecular formula C_34_H_52_O_8_, but they eluted at different time and are both present in extracts *man-7* and *man-15*. These isomers could be related to fusapirone (**5**; C_34_H_54_O_9_) a compound with antifungal activity, previously, isolated from *Fusarium semitectum* [62], it possess multiple chiral centers and can derived into compound (**5a**; C_34_H_52_O_8_) by dehydration (Figure 5).

**Figure 6:**
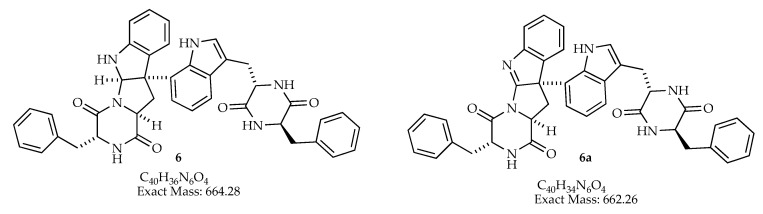
Nitrogen derivative **6a**, that could be present in extracts *man-7*, *man-9*, and *man-15.*

The dehydrogenation of asperacine (**6**; C_40_H_36_N_6_O_4_) results in compound **6a** (C_40_H_36_N_6_O_4_) with an imine function (Figure 6), this molecular formula was determined for extracts *man-7*, *man-9*, and *man-15*.

## 5. Conclusions

For this study were selected two strains of *Pestalotiopsis* endophytic fungi (*P. microspora* Hd18 and *P. mangiferae*), that are capable of producing secondary metabolites with relevant biological activities. The strategy developed in this work included variations of the culture conditions, the determination of the antibacterial activity of the obtained extracts, together with the effective analysis of the chemical profile using LC-HRMS. This strategy could improve the discovery of new molecules with a pharmaceutical potential, in this case antibacterial. Hence, this work confirmed changes in the chemical diversity and biological activity of *P. microspora* Hd18 and, principally, *P. mangiferae* Hd08 under varying the culture conditions.

Taking into account the chemical diversity and the preliminary antibacterial activity displayed by *P. mangiferae,* further work will need to establish and confirm the chemical composition of each extract as well as the antibacterial activity of a single compound.

## Figures and Tables

**Table 1:** Antibacterial activity in the disk diffusion test ^1^ of organic extracts produced by *Pestalotiopsis* spp.

Fungi	Extract	*E. coli*	*P. aeruginosa*	*S. tiphy*	*S. flexneri*	*P. vulgaris*	*S. enterica*	*L. pneumophIlla*	*E. faecalis*	*E. cloacae*	*P. multocida*	*K. rhizophila*	*L. monocytogenes*	*B. cereus*	*S. oralis*
*P. mangiferae*	*man-1*												8.5		9.5
	*man-2*		8.5			8.5							8.0		
	*man-3*		8.5		7.5				9.0	9.0			7.5		
	*man-6*		10.0	8.5	9.0		9.0				8.0		11.0	8.0	
	*man-7*							8.0	8.5	8.5		9.0			7.5
	*man-9*		12.5			8.5		8.5	8.0			7.5	7.5		
	*man-15*		9.5												
*P. microspora*	*mic-9*		8.0	8.5		7.5						8.0	7.5	8.5	
	*mic-10*	8.0										8.5	8.5		
	*mic-11*		9.5												
	*mic-12*		9.5			7.5								7.5	
Positive control	Gentamycin sulfate (10 μg/mL)	18.5	17.5	14.5	13.5	13.0	13.0	12.0	10.0	9.5	9.5	30.0	18.0	17.0	9.0

^1^ Results are given in mm of inhibition.

**Table 2:** Molecular ions of secondary metabolites present in extracts obtained from *P. mangiferae.*

Retention Time ^1^ (*t*_R_)	*m/z*	[M + H]^+^	[M + Na]^+^	Dimers	Molecular Formula	Extracts
19.44–19.50	588.36567	589.369		1177.720 [2M + H]^+^	C_34_H_52_O_8_	*man-7*, *man-15*
20.14–20.37	278.18765	279.194			C_17_H_26_O_3_	*man-7*
20.78–20.87	588.36567	589.369		1177.720 [2M + H]^+^	C_34_H_52_O_8_	*man-7*, *man-15*
21.55–21.59	282.18524	283.193		565.375 [2M + H]^+^	C_16_H_26_O_4_	*man-7*, *man-9*, *man-15*
	400.20917	401.217	423.199	801.424 [2M + H]^+^ 823.407 [2M + Na]^+^	C_20_H_32_O_8_	*man-7*, *man-9*, *man-15*
22.39–22.43	394.28663	395.295			C_27_H_38_O_2_	*man-7*, *man-15*
	412.29317	413.303			C_22_H_40_N_2_O_5_	*man-7*, *man-15*
	470.33906	471.347			C_30_H_46_O_4_	*man-7*, *man-15*
	488.34560	489.354		995.716 [2M + H_2_O + H]^+^	C_30_H_48_O_5_	*man-7*, *man-15*
23.40–23.45	314.24516	315.256	337.236		C_38_H_34_O_4_	*man-15*
23.72–23.81	428.30737		451.298		C_31_H_40_O	*man-7*, *man-15*
	428.31324	429.320			C_24_H_44_O_6_	*man-15*
24.54	278.22403	279.231	301.211	557.446 [2M + H]^+^	C_18_H_30_O_2_	*man-7*, *man-15*
24.60	452.31324		475.304	927.610 [2M + Na]^+^	C_26_H_44_O_6_	*man-15*
26.86	281.27132	282.279	304.261		C_18_H_35_NO	*man-7*, *man-15*
27.52	283.28697	284.294	306.276		C_18_H_37_NO	*man-7*, *man-15*
27.83–27.87	390.27646	391.283	413.261	803.536 [2M + Na]^+^	C_24_H_38_O_4_	*man-7*, *man-9*, *man-15*
28–33	337.33392	338.341	360.320	675.670 [2M + H]^+^	C_22_H_43_NO	*man-9*, *man-15*
28.40	418.28663	419.308	441.288	859.578 [2M + Na]^+^	C_29_H_38_O_2_	*man-7*
31.25–31.36	662.43884	663.444	685.40		C_40_H_36_NO_4_	*man-7*, *man-9*, *man-15*
	721.51233	722.519			C_41_H_71_NO_9_	*man-15*

^1^ Time is given in minutes.

## Supplementary Materials

The following are available online at https://www.mdpi.com/2309-608X/6/3/140/s1, Figure S1: Structure of compounds previously isolated from *P. mangiferae*. Table S1: Culture parameters and amount of organic extract produced by *Pestalotiopsis* spp., Table S2: Molecular ions and formulas of compounds isolated from the genus *Pestalotiopsis*
