# Supplementary material for: Exploring the Antibacterial Activity of Pestalotiopsis spp. under Different Culture Conditions and Their Chemical Diversity Using LC–ESI–Q–TOF–MS
Source: J Fungi (Basel). 2020 Aug 19;6(3):140. doi: 10.3390/jof6030140 (PMC7557868; doi:10.3390/jof6030140)
Supplement: Supplementary file 1 [file jof-06-00140-s001.pdf]

Supplementary Materials

Exploring the Antibacterial Activity of *Pestalotiopsis* spp. under Different Culture Conditions and Their Chemical Diversity Using LC–ESI–Q–TOF–MS

Madelaine M. Aguilar-Pérez [1,†], Daniel Torres-Mendoza [1,2,†], Roger Vásquez [1], Nivia Rios [3], and Luis Cubilla-Rios [1,*]

[1] Laboratory of Tropical Bioorganic Chemistry, Faculty of Natural, Exact Sciences and Technology, University of Panama, Panama 0824, Panama; mad25aguilar@gmail.com (M.M.A.-P.); dtorresm.507@gmail.com (D.T.-M.); royi071123@gmail.com (R.V.)

[2] Vicerrectoría de Investigación y Postgrado, University of Panama, Panama 0824, Panama

[3] Department of Microbiology, Faculty of Natural, Exact Sciences and Technology, University of Panama, Panama 0824, Panama; toxogondii@gmail.com

* Correspondence: luis.cubilla@up.ac.pa; Tel.: +507-6676-5824

† These authors contributed equally to this work.

Received: 16 July 2020; Accepted: 17 August 2020; Published: 19 August 2020

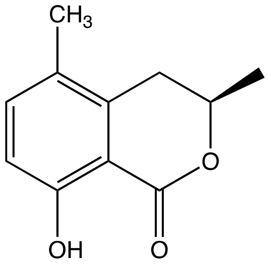

$C_{11}H_{12}O_3$ **(S1)**
Exact Mass: 192.08

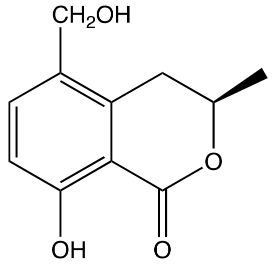

$C_{11}H_{12}O_4$ **(S2)**
Exact Mass: 208.07

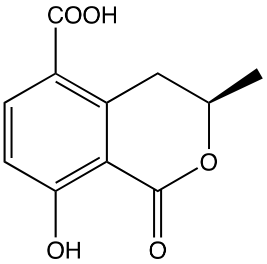

$C_{11}H_{10}O_5$ **(S3)**
Exact Mass: 222.05

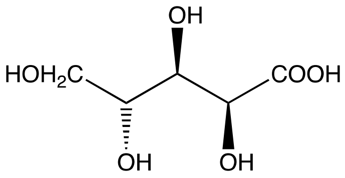

$C_5H_{10}O_6$ **(S4)**
Exact Mass: 166.05

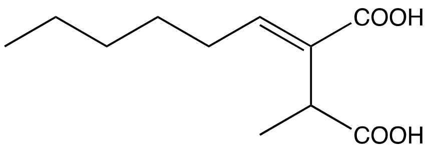

$C_{11}H_{18}O_4$ **(S5)**
Exact Mass: 214.12

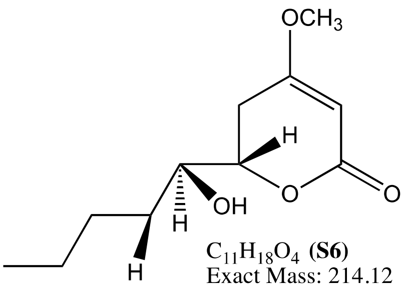

$C_{11}H_{18}O_4$ **(S6)**
Exact Mass: 214.12

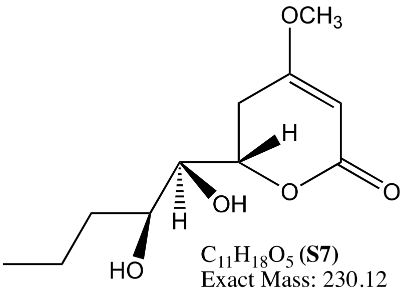

$C_{11}H_{18}O_5$ **(S7)**
Exact Mass: 230.12

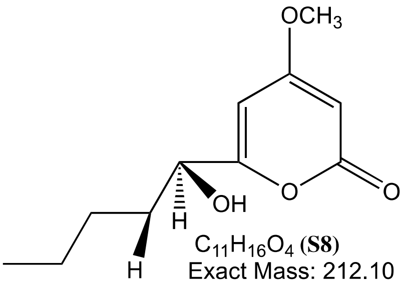

$C_{11}H_{16}O_4$ **(S8)**
Exact Mass: 212.10

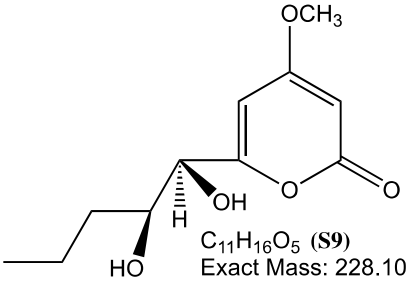

$C_{11}H_{16}O_5$ **(S9)**
Exact Mass: 228.10

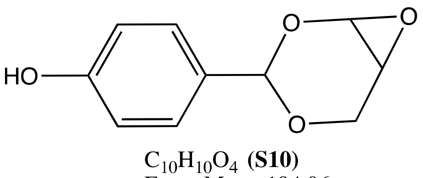

$C_{10}H_{10}O_4$ **(S10)**
Exact Mass: 194.06

Figure S1: Structure compounds previously isolated from *P. mangiferae*. Compounds **S1**-**S9** were reported by Ortega et. al (2014) and compound **S10** by Subban et. al (2013).

Table S1. Culture parameters and amount of organic extract produced by *Pestalotiopsis* spp.

		Culture parameters			Amount of the organic extract (mg)			
**Phase**	**Variation**	**Chemicals**	**pH**	**Temp. (°C)**	***P. mangiferae***		***P. microspore***	
I	Chemicals	Arg	4.6 *	26	*man-1*	188.0	*mic-1*	86.0
		Glu			*man-2*	118.0	*mic-2*	100.0
		$FeSO_4$			*man-3*	256.0	*mic-3*	251.0
		$CaCl_2$			*man-4*	232.0	*mic-4*	297.0
		$CuSO_4$			*man-5*	160.0	*mic-5*	329.0
II	pH	$CaCl_2$	4.0	26	*man-6*	15.4	*mic-6*	157.0
			4.6		*man-7*	15.2	*mic-7*	127.1
			5.6		*man-8*	15.0	*mic-8*	32.7
		$CuSO_4$	4.0		*man-9*	264.3	*mic-9*	260.0
			4.6		*man-10*	52.9	*mic-10*	73.4
			5.6		*man-11*	67.8	*mic-11*	50.4
III	Incubation temperature	$CaCl_2$	4.0	24	*man-12*	262.0	*mic-12*	183.0
				28	*man-13*	44.0	*mic-13*	306.0
				30	*man-14*	528.0	*mic-14*	148.0
		$CuSO_4$		24	*man-15*	62.0	*mic-15*	1194.0
				28	*man-16*	278.0	*mic-16*	1012.0
				30	*man-17*	448.0	*mic-17*	249.0

*** pH of the commercial Malt Extract Agar culture media**

Table S2. Molecular ions and formulas of compounds isolated from the genus *Pestalotiopsis*

Compound	Formula	Molecular ion *m/z*	Source	Reference
Ficifuranone B	$C_8H_{10}O_4$	171.0655 $[M + H]^+$	*P. fici*	[1]
Ficifuranone A	$C_9H_{12}O_4$	185.0811 $[M + H]^+$	*P. fici*	[1]
4-(2, 4, 7-trioxa-bicyclo[4.1.0]heptan-e-yl)phenol	$C_{10}H_{11}O_4$	195.0657 $[M + H]^+$	*P. mangiferae*	[2]
Pestaolide	$C_{12}H_{20}O_2$	196.1463 $[M]^+$	*P.* sp. PSU-MA69	[3]
Pestalotiopyrone N	$C_{10}H_{29}O_5$	209.0455 $[M - H]^-$	*P. neglecta* SCSI41403	[4]
6-[(7S, 8R)-8-propyloxiran-1-yl]-4-methoxy-pyran-2-one	$C_{11}H_{15}O_4$	211.0965 $[M + H]^+$	*P. versicolor*	[5]
Pestatheranone A	$C_{12}H_{19}O_3$	211.1329 $[M + H]^+$	*P. theaea*	[6]
Pestalofuranone A	$C_{11}H_{14}O_3Na$	217.0835 $[M + Na]^+$	*P. besseyi*	[7]
Pestalofuranone B	$C_{11}H_{14}O_3Na$	217.0835 $[M + Na]^+$	*P. besseyi*	[7]
Pestalofuranone D	$C_{11}H_{14}O_3Na$	217.0835 $[M + Na]^+$	*P. besseyi*	[7]
Pestalactam E	$C_{10}H_{13}NO_3Na$	218.0788 $[M + Na]^+$	*P.* sp.	[8]
Pestalofuranone C	$C_{11}H_{16}O_3Na$	219.0992 $[M + Na]^+$	*P. besseyi*	[7]
Pestalofuranone E	$C_{11}H_{16}O_3Na$	219.0992 $[M + Na]^+$	*P. besseyi*	[7]
4, 6-dihydroxy-7-formyl-3-methylcoumarin	$C_{11}H_9O_5$	221.0444 $[M + H]^+$	*P. versicolor*	[5]
Pestalochromone C	$C_{11}H_{11}ClO_3$	226.0391 $[M]^+$	*P.* sp. PSU-MA69	[3]
Microsporaline C	$C_{12}H_{14}O_3Na$	229.0835 $[M + Na]^+$	*P. microspora* SC3082	[9]
Heterocornol C	$C_{12}H_{14}O_3Na$	229.0841 $[M + Na]^+$	*P. heterocornis*	[10]
Pestaloficiol S	$C_{15}H_{17}O_2$	229.1223 $[M + H]^+$	*P. fici*	[11]
Pestalactam D	$C_{10}H_{13}{}^{35}ClNO_3$	230.0584 $[M + H]^+$	*P.* sp.	[8]
Pestalotioquinol C	$C_{13}H_{11}O_4$	231.0663 $[M - H]^-$	*P. neglecta*	[12]
Pestalotioquinol D	$C_{13}H_{11}O_4$	231.0663 $[M - H]^-$	*P. neglecta*	[12]
Pestalrone A	$C_{11}H_{19}O_5$;<br>$C_{11}H_{18}O_5Na$	231.1227 $[M + H]^+$;<br>253.1046 $[M + Na]^+$	*P. zonata*	[13]
Pestallic acid G	$C_{13}H_{14}NO_3$	232.0968 $[M + H]^+$	*P. neglecta* SCSI41403	[4]
Isopolisin B	$C_{12}H_{18}O_3Na$	233.1154 $[M + Na]^+$	*P. heterocornis*	[14]
Pestalpyrone D	$C_{12}H_{13}O_5$	237.0757 $[M + H]^+$	*P.* sp.	[15]
Pestalpyrone E	$C_{12}H_{13}O_5$	237.0757 $[M + H]^+$	*P.* sp.	[15]
Pestaloisocoumarin A	$C_{12}H_{13}O_5$	237.0763 $[M - H]^-$	*P. heterocornis*	[14]
Pestalrone B	$C_{11}H_{18}O_4Na$	237.1088 $[M + Na]^+$	*P. karstenii*	[16]
Foedanolide	$C_{14}H_{21}O_3$	237.1485 $[M + H]^+$	*P. foedan*	[17]

Pestalotiol A	$C_{11}H_{20}O_4Na$	239.1259 $[M + Na]^+$	*P. heterocornis*	[14]
Heterocornol D	$C_{12}H_{12}O_4Na$	243.0633 $[M + Na]^+$	*P. heterocornis*	[10]
Pestalotiopamide E	$C_{11}H_{18}NO_5$	244.1185 $[M + H]^+$	*P.* sp.	[18]
Vaccinol N	$C_{12}H_{14}O_4Na$	245.0784 $[M + Na]^+$	*P. vaccinii*	[19]
Pestalotiopisorin B	$C_{12}H_{14}O_4Na$	245.0790 $[M + Na]^+$	*P.* sp HHL101	[20]
Vaccinol L	$C_{12}H_{16}O_4Na$	247.0941 $[M + Na]^+$	*P. vaccinii*	[19]
Heterocornol A	$C_{12}H_{16}O_4Na$	247.0946 $[M + Na]^+$	*P. heterocornis*	[10]
4, 10-dihydroxy-gamahorin	$C_{12}H_{14}O_6Na$	249.1097 $[M + Na]^+$	*P.* sp. M-23	[21]
Vaccinol M	$C_{12}H_{18}O_4Na$	249.1097 $[M + Na]^+$	*P. vaccinii*	[19]
Pestalofíciol R	$C_{13}H_{15}O_5$	251.0914 $[M + H]^+$	*P. fici*	[11]
Disseminin D (9, 10-*cis*)	$C_{13}H_{24}O_3Na$	251.1623 $[M + Na]^+$	*P. disseminata*	[22]
Disseminin E (9, 10-*trans*)	$C_{13}H_{24}O_3Na$	251.1623 $[M + Na]^+$	*P. disseminata*	[22]
Pestaisocoumarin A	$C_{12}H_{12}O_6$	253.0706 $[M + H]^+$	*P. heterocornis*	[23]
Photipyrone B	$C_{11}H_{18}O_5Na$	253.1040 $[M + Na]^+$	*P. photiniae*	[24]
Photipyrone C	$C_{11}H_{18}O_5Na$	253.1043 $[M + Na]^+$	*P. photiniae*	[24]
Pestalotioprolide G	$C_{14}H_{21}O_4$	253.1434 $[M + H]^+$	*P. microspora*	[25]
Spiciferone D	$C_{14}H_{23}O_4$	255.1596 $[M + H]^+$	*P. disseminata*	[22]
Spiciferone E	$C_{14}H_{23}O_4$	255.1596 $[M + H]^+$	*P. disseminata*	[22]
Pestalol D	$C_{14}H_{18}O_3Na$	257.1153 $[M + Na]^+$	*P.* sp. AcBC2	[26]
Pestalotioprolide C	$C_{14}H_{25}O_4$	257.1747 $[M + H]^+$	*P. microspora*	[25]
Microsporaline A	$C_{12}H_{14}O_5Na$	261.0733 $[M + Na]^+$	*P. microspora* SC3082	[9]
Microsporaline B	$C_{12}H_{16}O_5Na$	263.0890 $[M + Na]^+$	*P. microspora* SC3082	[9]
Pestallic acid F	$C_{14}H_{15}O_5$	263.0925 $[M - H]^-$	*P. neglecta* SCSI41403	[4]
Pestalactam F	$C_{11}H_{15}NO_5Na$	264.0842 $[M + Na]^+$	*P.* sp.	[8]
Pestalotiorin	$C_{14}H_{16}O_5$	264.0992 $[M]^+$	*P.* sp. PSU-ES194	[27]
Pestalactone C	$C_{13}H_{13}O_6$	265.0707 $[M + H]^+$	*P.* sp.	[15]
Disseminin A	$C_{13}H_{22}O_4Na$	265.1416 $[M + Na]^+$	*P. disseminata*	[22]
Disseminin B	$C_{13}H_{22}O_4Na$	265.1416 $[M + Na]^+$	*P. disseminata*	[22]
Ficipyrone B	$C_{14}H_{18}O_5$	267.1224 $[M + H]^+$	*P. fici*	[1]
Disseminin C	$C_{13}H_{24}O_4Na$	267.1572 $[M + Na]^+$	*P. disseminata*	[22]
Pestalotioprolide H	$C_{15}H_{23}O_4$	267.1591 $[M + H]^+$	*P. microspora*	[25]
2 -hydroxy-7 , 8 -epoxy-isodrimeninol	$C_{15}H_{24}O$	268.1675 $[M]^+$	*P.* sp. M-23	[21]
Pestalotioprolide E	$C_{14}H_{24}O_4N$	270.1700 $[M + NH_4]^+$	*P. microspora*	[25]
Pestalotioprolide F	$C_{14}H_{24}O_4N$	270.1700 $[M + NH_4]^+$	*P. microspora*	[25]

Heterocornol M	$C_{15}H_{20}O_3Na$	271.1310 [M + Na]$^+$	*P. heterocornis XWS03F09*	[28]
Ficipyrone A	$C_{14}H_{22}O_5$	271.1535 [M + H]$^+$	*P. fici*	[1]
Pestalotionol	$C_{14}H_{18}O_3K$	273.0893 [M + K]$^+$	*P.* sp. PSU-ES194	[27]
Vaccinol K	$C_{14}H_{18}O_4Na$	273.1097 [M + Na]$^+$	*P. vaccinii*	[19]
Heterocornol B	$C_{14}H_{18}O_4Na$	273.1103 [M + Na]$^+$	*P. heterocornis*	[10]
Heterocornol N	$C_{14}H_{18}O_4Na$	273.1103 [M + Na]$^+$	*P. heterocornis XWS03F09*	[28]
11-dehydro-3 -hydroxyisodrimeninol	$C_{15}H_{22}O_3Na$	273.1461 [M + Na]$^+$	*P.* sp. M-23	[21]
Heterocornol G	$C_{17}H_{21}O_3$	273.1491 [M - H]$^-$	*P. heterocornis*	[10]
Pestalochromone A	$C_{12}H_{17}ClO_5$	276.0759 [M]$^+$	*P.* sp. PSU-MA69	[3]
Pestalochromone B	$C_{12}H_{17}ClO_5$	276.0759 [M]$^+$	*P.* sp. PSU-MA69	[3]
Neopestalone	$C_{14}H_{13}O_6$	277.0707 [M + H]$^+$	*P. neglecta* SCSI41403	[4]
Pestaloisocoumarin B	$C_{14}H_{15}O_6$	279.0869 [M - H]$^-$	*P. heterocornis*	[14]
Pestalactone A	$C_{14}H_{17}O_6$	281.1020 [M + Na]$^+$	*P.* sp.	[15]
Heterocornol O	$C_{15}H_{18}O_4Na$	285.1103 [M + Na]$^+$	*P. heterocornis XWS03F09*	[28]
Heterocornol P	$C_{15}H_{18}O_4Na$	285.1103 [M + Na]$^+$	*P. heterocornis XWS03F09*	[28]
Microsporaline D	$C_{14}H_{16}O_5Na$	287.0890 [M + Na]$^+$	*P. microspora* SC3082	[9]
7--methylnigrosporolide	$C_{15}H_{22}O_4Na$	289.1410 [M + Na]$^+$	*P. microspora*	[25]
Pestalotioprolide D	$C_{15}H_{22}O_4Na$	289.1410 [M + Na]$^+$	*P. microspora*	[25]
Pestalotioprolide B	$C_{14}H_{20}O_5Na$	291.1203 [M + Na]$^+$	*P. microspora*	[25]
2 , 8 -dihydroxy-6, 7-en-isodrimeninol	$C_{15}H_{24}O_4Na$	291.1567 [M + Na]$^+$	*P.* sp. M-23	[21]
Pestaloporonin B	$C_{15}H_{24}O_4Na$	291.1572 [M + Na]$^+$	*P.* sp.	[29]
Heterocornol I	$C_{17}H_{23}O_4$	291.1596 [M - H]$^-$	*P. heterocornis*	[10]
Heterocornol J	$C_{17}H_{23}O_4$	291.1596 [M - H]$^-$	*P. heterocornis*	[10]
Heterocornol K	$C_{17}H_{23}O_4$	291.1596 [M - H]$^-$	*P. heterocornis*	[10]
Vaccinol J	$C_{17}H_{20}O_3Na$	295.1305 [M + Na]$^+$	*P. vaccinii*	[19]
Pestaloporonin A	$C_{16}H_{23}O_5$	295.1545 [M - H]$^-$	*P.* sp.	[29]
Heterocornol H	$C_{17}H_{24}O_3Na$	299.1623 [M + Na]$^+$	*P. heterocornis*	[10]
Pestalol E	$C_{13}H_{17}O_6S$	301.0745 [M + H]$^+$	*P.* sp. AcBC2	[26]
Pestauvicolactone A	$C_{16}H_{16}NO_5$	302.1023 [M + H]$^+$	*P. uvicola*	[30]
Pestalactone B	$C_{13}H_{14}O_7Na$	305.0632 [M + Na]$^+$	*P.* sp.	[15]
Vaccinol R	$C_{17}H_{23}O_5$	307.1540 [M + H]$^+$	*P. vaccinii*	[19]

Vaccinol S	$C_{17}H_{23}O_5$	307.1540 [M + H]$^+$	*P. vaccinii*	[19]
Pestalotioquinol E	$C_{21}H_{25}O_2$	309.1860 [M - H]$^-$	*P. neglecta*	[12]
Pestalol A	$C_{19}H_{28}O_2Na$	311.1987 [M + Na]$^+$	*P.* sp. AcBC2	[26]
Vaccinol O	$C_{17}H_{22}O_4Na$	313.1410 [M + Na]$^+$	*P. vaccinii*	[19]
Heterocornol F	$C_{17}H_{24}O_4Na$	315.1572 [M + Na]$^+$	*P. heterocornis*	[10]
Pestalol B	$C_{19}H_{26}O_3Na$	325.1779 [M + Na]$^+$	*P.* sp. AcBC2	[26]
Pestaloxanthone	$C_{17}H_{14}O_7$	330.0734 [M]$^+$	*P.* sp. PSU-MA69	[3]
Vaccinol P	$C_{17}H_{24}O_5Na$	331.1516 [M + Na]$^+$	*P. vaccinii*	[19]
14-acetylhumulane	$C_{17}H_{24}O_5Na$	331.1516 [M + Na]$^+$	*P.* sp.	[31]
Pestaloporinate F	$C_{17}H_{26}O_5Na$	333.1672 [M + Na]$^+$	*P.* sp.	[31]
(10*S*)-12, 16-epoxy-17(15→16)-abeo-3, 5, 8, 12, 15-abietapentaen-2, 7, 11, 14-tetraone	$C_{20}H_{15}O_5^-$	335.0919 [M - H]$^-$	*P. adusta*	[32]
Pestalotic acid D	$C_{18}H_{23}ClO_4$	338.1285 [M]$^+$	*P.* sp. cr014	[33]
Pestalofíciol Q	$C_{18}H_{24}O_5Na$	343.1516 [M + Na]$^+$	*P. fici*	[11]
Pestalol C	$C_{19}H_{28}O_4Na$	343.1885 [M + Na]$^+$	*P.* sp. AcBC2	[26]
Pestalotioquinol B	$C_{21}H_{27}O_4$	343.1909 [M - H]$^-$	*P. microspora*	[34]
Pestalotione A	$C_{17}H_{13}O_8$	345.0605 [M + H]$^+$	*P. theae*	[35]
Pestalotic acid B	$C_{19}H_{21}O_6$	345.1338 [M - H]$^-$	*P.* sp. cr014	[33]
Pestalic acid B	$C_{18}H_{26}O_5Na$	345.1672 [M + Na]$^+$	*P.* sp. FT172	[36]
Pestalic acid D	$C_{18}H_{26}O_5Na$	345.1672 [M + Na]$^+$	*P.* sp. FT172	[36]
Pestaloporinate C	$C_{18}H_{26}O_5Na$	345.1672 [M + Na]$^+$	*P.* sp.	[31]
Pestaloporinate D	$C_{18}H_{26}O_5Na$	345.1672 [M + Na]$^+$	*P.* sp.	[31]
Dechloromaldoxin	$C_{17}H_{15}O_8$	347.0761 [M + H]$^+$	*P. fici*	[37]
Pestaloporinate G	$C_{18}H_{28}O_5Na$	347.1829 [M + Na]$^+$	*P.* sp.	[31]
Pestalotic acid A	$C_{19}H_{25}O_6$	349.1651 [M - H]$^-$	*P.* sp. cr014	[33]
Pestalotione B	$C_{19}H_{19}O_7$	359.1125 [M + H]$^+$	*P. theae*	[35]
Pestaloporinate B	$C_{18}H_{24}O_6Na$	359.1465 [M + Na]$^+$	*P.* sp.	[31]
Pestaloether B	$C_{16}H_{15}ClO_6Na$	361.0455 [M + Na]$^+$	*P.* sp. PSU-MA69	[3]
Pestalic acid C	$C_{18}H_{26}O_6Na$	361.1622 [M + Na]$^+$	*P.* sp. FT172	[36]
Cytosporin M	$C_{19}H_{30}O_5Na$	361.1970 [M + Na]$^+$	*P.* sp. IQ-011	[38]
2′-aminodechloromaldoxin	$C_{17}H_{16}NO_8$	362.0876 [M + H]$^+$	*P. flavidula*	[39]
2′-aminodechlorogeodoxin	$C_{17}H_{16}NO_8$	362.0876 [M + H]$^+$	*P. flavidula*	[39]
Pestaloether D	$C_{18}H_{18}O_8$	362.0996 [M]$^+$	*P.* sp. PSU-MA69	[3]
Vaccinol Q	$C_{12}H_{11}O_3Na$	363.1778 [M + Na]$^+$	*P. vaccinii*	[19]
Pestalotioquinol A	$C_{21}H_{28}O_4Na$	367.1885 [M + Na]$^+$	*P. microspora*	[34]

Sinopestalotiollide A	$C_{21}H_{21}O_6$	369.1333 [M - H]$^-$	*P. palmarum*	[40]
Sinopestalotiollide B	$C_{21}H_{21}O_6$	369.1333 [M - H]$^-$	*P. palmarum*	[40]
Pestynol	$C_{21}H_{30}O_4Na$	369.2042 [M + Na]$^+$	*P. humus*	[41]
Ambuic acid derivative 4	$C_{19}H_{24}O_6Na$	371.1463 [M + Na]$^+$	*P.* sp.	[42]
Pestalotic acid F	$C_{19}H_{24}O_6Na$	371.1471 [M + Na]$^+$	*P.* sp. cr014	[33]
Heterocornol L	$C_{19}H_{24}O_6Na$	371.1471 [M + Na]$^+$	*P. heterocornis*	[10]
Sinopestalotiollide C	$C_{21}H_{23}O_6$	371.1489 [M - H]$^-$	*P. palmarum*	[40]
Pestathenol A	$C_{19}H_{28}O_6Na$	375.1778 [M + Na]$^+$	*P. theae*	[6]
Pestathenol B	$C_{19}H_{28}O_6Na$	375.1778 [M + Na]$^+$	*P. theae*	[6]
Ambuic acid derivative 3	$C_{19}H_{28}O_6Na$	375.1778 [M + Na]$^+$	*P.* sp.	[42]
Ambuic acid derivative 5	$C_{19}H_{28}O_6Na$	375.1778 [M + Na]$^+$	*P. neglecta*	[43]
Ambuic acid derivative 6	$C_{19}H_{28}O_6Na$	375.1778 [M + Na]$^+$	*P.* sp.	[42]
Pestalotic acid C	$C_{18}H_{25}ClO_5Na$	379.1288 [M + Na]$^+$	*P.* sp. cr014	[33]
Pestalic acid E	$C_{19}H_{26}O_7Na$	385.1571 [M + Na]$^+$	*P.* sp. FT172	[36]
Ambuic acid derivative 2	$C_{19}H_{24}O_7Na$	387.14141 [M + Na]$^+$	*P.* sp.	[42]
Ambuic acid derivative 2	$C_{19}H_{24}O_7Na$	387.1414 [M + Na]$^+$	*P. neglecta*	[43]
Virgatolide C	$C_{19}H_{24}O_7Na$	387.1420 [M + Na]$^+$	*P. virgatula*	[44]
Virgatolide B	$C_{19}H_{24}O_7Na$	387.1421 [M + Na]$^+$	*P. virgatula*	[44]
Pitholide E	$C_{22}H_{27}O_6$	387.1808 [M + H]$^+$	*P. microspora*	[45]
Pestalotioquinoside C	$C_{22}H_{27}O_6$	387.1813 [M - H]$^-$	*P. neglecta*	[12]
Ambuic acid derivative 1	$C_{19}H_{26}O_7Na$	389.1571 [M + Na]$^+$	*P. neglecta*	[43]
Polyketide-terpene hybrid metabolite 1	$C_{19}H_{26}O_7Na$	389.1576 [M + Na]$^+$	*P.* sp.	[46]
Pestalotic acid G	$C_{19}H_{26}O_7Na$	389.1576 [M + Na]$^+$	*P.* sp. cr014	[33]
Pestalotic acid H	$C_{19}H_{26}O_7Na$	389.1576 [M + Na]$^+$	*P.* sp. cr014	[33]
Pestalotic acid I	$C_{19}H_{26}O_7Na$	389.1576 [M + Na]$^+$	*P.* sp. cr014	[33]
Ambuic acid derivative 1	$C_{19}H_{26}O_7Na$	389.1576 [M + Na]$^+$	*P.* sp.	[46]
Ambuic acid derivative 5	$C_{19}H_{26}O_7Na$	389.1576 [M + Na]$^+$	*P.* sp.	[46]
Polyketide-terpene hybrid metabolite 5	$C_{19}H_{26}O_7Na$	389.1576 [M + Na]$^+$	*P.* sp.	[46]
Pestalpolyol E	$C_{22}H_{38}O_4Na$	389.2668 [M + Na]$^+$	*P.* sp. PG52	[47]
Pestalone E	$C_{20}H_{20}{}^{35}ClO_6$	391.0948 [M + H]$^+$	*P. neglecta*	[48]
Pestaloporinate E	$C_{20}H_{32}O_6Na$	391.2091 [M + Na]$^+$	*P.* sp.	[31]
Ambuic acid derivative 3	$C_{19}H_{30}O_7Na$	393.1884 [M + Na]$^+$	*P. neglecta*	[43]
Pestalotioquinol F	$C_{22}H_{28}O_5Na$	395.1829 [M + Na]$^+$	*P. neglecta*	[12]
Pestaloether A	$C_{18}H_{17}ClO_8$	396.0612 [M]$^+$	*P.* sp. PSU-MA69	[3]
Pestaloether C	$C_{18}H_{17}ClO_8$	396.0612 [M]$^+$	*P.* sp. PSU-MA69	[3]

Sinopestalotiollide D	$C_{22}H_{23}O_7$	399.1438 $[M - H]^-$	*P. palmarum*	[40]
Pestalone F	$C_{21}H_{22}{}^{35}ClO_6$	405.1105 $[M + H]^+$	*P. neglecta*	[48]
Pestaloporinate A	$C_{20}H_{30}O_7Na$	405.1884 $[M + Na]^+$	*P.* sp.	[31]
Ambuic acid derivative 5	$C_{22}H_{30}O_6Na$	413.1935 $[M + Na]^+$	*P.* sp.	[42]
Pestalotic acid E	$C_{21}H_{26}O_7Na$	413.2668 $[M + Na]^+$	*P.* sp. cr014	[33]
Ambuic acid derivative 1	$C_{21}H_{28}O_7Na$	415.1719 $[M + Na]^+$	*P.* sp.	[42]
Ambuic acid derivative 4	$C_{21}H_{30}O_7Na$	417.1884 $[M + Na]^+$	*P. neglecta*	[43]
Pestazonatic acid	$C_{20}H_{19}O_{10}$	419.0984 $[2M - H]^-$	*P. zonata*	[13]
(±)-Pestalachloride D	$C_{21}H_{20}Cl_2O_5$	422.0682 $[M]^+$	*P.* sp.	[49]
Pestalpolyol B	$C_{25}H_{42}O_4Na$	429.2981 $[M + Na]^+$	*P.* sp. cr013	[50]
Pestalic acid A	$C_{21}H_{28}O_8Na$	431.1676 $[M + Na]^+$	*P.* sp. FT172	[36]
Polyketide-terpene hybrid metabolite 2	$C_{21}H_{28}O_8Na$	431.1682 $[M + Na]^+$	*P.* sp.	[46]
Polyketide-terpene hybrid metabolite 4	$C_{21}H_{28}O_8Na$	431.1682 $[M + Na]^+$	*P.* sp.	[46]
Ambuic acid derivative 2	$C_{21}H_{28}O_8Na$	431.1682 $[M + Na]^+$	*P.* sp.	[46]
Ambuic acid derivative 4	$C_{21}H_{28}O_8Na$	431.1682 $[M + Na]^+$	*P.* sp.	[46]
Polyketide-terpene hybrid metabolite 3	$C_{21}H_{30}O_8Na$	433.1838 $[M + Na]^+$	*P.* sp.	[46]
Ambuic acid derivative 3	$C_{21}H_{30}O_8Na$	433.1838 $[M + Na]^+$	*P.* sp.	[46]
Pestalone C	$C_{20}H_{20}{}^{79}BrO_6$<br>$C_{20}H_{20}{}^{81}BrO_6$	435.0443 $[M + H]^+$;<br>437.0423 $[M + H]^+$	*P. neglecta*	[48]
Pestalone B	$C_{21}H_{22}{}^{79}BrO_6$<br>$C_{21}H_{22}{}^{81}BrO_6$	449.0600 $[M + H]^+$;<br>451.0579 $[M + H]^+$	*P. neglecta*	[48]
Pestalotioquinoside A	$C_{27}H_{35}O_6$	455.2439 $[M - H]^-$	*P. neglecta*	[12]
Virgatolide A	$C_{22}H_{26}O_9Na$	457.1466 $[M + Na]^+$	*P. virgatula*	[44]
Pestalpolyol H	$C_{28}H_{48}O_4Na$	471.3450 $[M + Na]^+$	*P.* sp. PG52	[47]
Pestalpolyol A	$C_{28}H_{50}O_4Na$	473.3607 $[M + Na]^+$	*P.* sp. cr013	[50]
(±)-Pestaloxazine A	$C_{22}H_{33}N_4O_8$	481.22984 $[M + H]^+$	*P.* sp. ZJ-2009-7-6	[51]
Pestalone G	$C_{21}H_{21}{}^{79}Br^{35}ClO_6$	483.0210 $[M + H]^+$	*P. neglecta*	[48]
Pestalone H	$C_{21}H_{21}{}^{79}Br^{35}ClO_6$	483.0210 $[M + H]^+$	*P. neglecta*	[48]
Pestaloamide A	$C_{24}H_{24}N_2O_5S_2$	485.1195 $[M + H]^+$	*P. sp.* HS30	[52]
Pestalpolyol G	$C_{28}H_{48}O_5Na$	485.3243 $[M + Na]^+$	*P.* sp. PG52	[47]
Pestalotione C	$C_{22}H_{24}O_{11}Na$	487.1216 $[M + Na]^+$	*P. theae*	[35]
Pestalotioquinoside B	$C_{27}H_{35}O_8$	487.2326 $[M + H]^+$	*P. neglecta*	[12]
Pestalpolyol F	$C_{28}H_{48}O_5Na$	487.3399 $[M + Na]^+$	*P.* sp. PG52	[47]
Pestaloamide B	$C_{24}H_{24}N_2O_5S_2$	507.1019 $[M + Na]^+$	*P. sp.* HS30	[52]
11 -acetoxyisoaustinone	$C_{27}H_{32}O_8Na$	507.1995 $[M + Na]^+$	*P.* sp. PSU-ES194	[27]

4 , 5 -diacetoxy-9 -benzoyloxy-7 H-eudesman-1 , 2 , 11, 14-tetraol	$C_{26}H_{37}O_{10}$	509.2381 $[M + H]^+$	*P.* sp.	[53]
7-hydroxydehydroaustin	$C_{27}H_{31}O_{10}$	515.1917 $[M + H]^+$	*P.* sp. PSU-ES194	[27]
Pestalone D	$C_{21}H_{21}{}^{79}BrO_6$	526.9705 $[M + H]^+$	*P. neglecta*	[48]
Dihydroberkleasmin A	$C_{30}H_{50}O_7Na$	545.3454 $[M + Na]^+$	*P. photiniae*	[54]
Pestalotiopen A	$C_{27}H_{33}ClO_{10}$	551.16840 $[M - H]^-$	*P.* sp.	[55]
Pestalpolyol C	$C_{33}H_{58}O_5Na$	557.4182 $[M + Na]^+$	*P.* sp. cr013	[50]
Pestalpolyol D	$C_{34}H_{60}O_5Na$	571.4338 $[M + Na]^+$	*P.* sp. cr013	[50]
Pestaloporonin C	$C_{32}H_{48}O_8Na$	583.3247 $[2 M + Na]^+$	*P.* sp.	[29]
1β, 5 , 6 , 14-tetraacetoxy-9 -benzoyloxy-7 H-eudesman-2 , 11-diol	$C_{30}H_{41}O_{12}$	593.2593 $[M + H]^+$	*P.* sp.	[53]
Chloropestolide G	$C_{32}H_{31}ClO_{10}Na$	633.1498 $[M + Na]^+$	*P. fici*	[37]
Chloropestolide D	$C_{33}H_{36}ClO_{11}$	643.1941 $[M + H]^+$	*P. fici*	[37]
Chloropestolide E	$C_{33}H_{36}ClO_{11}$	643.1941 $[M + H]^+$	*P. fici*	[37]
Chloropupukeanolide E	$C_{32}H_{33}ClO_{11}Na$	651.1604 $[M + Na]^+$	*P. fici*	[56]
Chloropestolide B	$C_{33}H_{35}ClO_{11}Na$	665.1760 $[M + Na]^+$	*P. fici*	[37]
Chloropestolide C	$C_{33}H_{35}ClO_{11}Na$	665.1760 $[M + Na]^+$	*P. fici*	[37]
Chloropestolide F	$C_{33}H_{35}ClO_{11}Na$	665.1760 $[M + Na]^+$	*P. fici*	[37]
Chloropupukeanolide C	$C_{33}H_{35}ClO_{11}Na$	665.1760 $[M + Na]^+$	*P. fici*	[56]
Chloropupukeanolide D	$C_{33}H_{35}ClO_{11}Na$	665.1760 $[M + Na]^+$	*P. fici*	[56]
Pestaloquinol B	$C_{38}H_{52}O_{10}Na$	691.3453 $[M + Na]^+$	*P.* sp.	[57]
Pestaloquinol A	$C_{38}H_{52}O_{10}Na$	691.3453 $[M + Na]^+$	*P.* sp.	[57]
Torreyanic acid analogue	$C_{38}H_{45}O_{12}$	693.2917 $[M - H]^-$	P. sp.	[42]
Pestalotiopen B	$C_{34}H_{45}ClO_{13}$	695.24704 $[M - H]^-$	*P.* sp.	[55]
Pestauvicomorpholine A	$C_{43}H_{53}NO_7Na$	718.3714 $[M + Na]^+$	*P. uvicola*	[30]
Pestiocandin	$C_{43}H_{62}O_{16}Na$	857.3936 $[M + Na]^+$	*P. humus*	[58]
1-undecene-2, 3-dicarboxylic acid	$C_{13}H_{22}O_4$	243.1591	*P. theae*	[59]
Oxopestalochromane	$C_{13}H_{13}O_6$	265.0710	*P.* sp. IQ-011	[38]
Chlorotheolide B	$C_{29}H_{32}ClO_{10}$	575.1679	*P. theae*	[59]
Chlorotheolide A	$C_{30}H_{36}ClO_{11}$	607.1941	*P. theae*	[59]
Pestalotione D	$C_{19}H_{20}O_8$	Not reported	*P. theae*	[35]
Cytosporin N	$C_{19}H_{28}O_6$	Not reported	*P.* sp. IQ-011	[38]
Cuautepestalorin	$C_{32}H_{36}O_{10}$	Not reported	*P.* sp. IQ-011	[38]
6-(1-hydroxypentyl)-4-methoxy-pyran-2-one		191 $[M + Na – CO_2]^+$; 213 $[M + H]^+$;	*P. guepinii*	[60]

		235 [M + Na]$^+$;<br>447 [2XM + Na]$^+$		
6-hydroxymethyl-4-methoxy-5,6-dihydro-2H-pyran-2-one	$C_7H_{10}O_4$	159.2 [M + H]$^+$	*P. sydowiana*	[61]
4-(hydroxymethyl)catechol	$C_7H_8O_3Na$	163.1 [M + Na]$^+$	*P.* sp.	[62]
(4*S*)-4, 8-dihydroxy-1-tetralone	$C_{10}H_{10}O_3$	179 [M + H]$^+$	*P.* sp. EJC07	[63]
Pestalopyrone	$C_{11}H_{12}O_3$	179.1 [M - H]$^-$;<br>181.1 [M + H]$^+$	*P. neglecta* SCSI41403	[4]
(3*R*, 4*R*, 6*R*, 7*S*)-7-hydroxyl-3, 7-dimethyl-oxabicyclo[3.3.1]-nonan-2-one	$C_{10}H_{16}O_3$	183 [M - H]$^-$	*P. foedan*	[64]
2,4-dihydroxy-3,5,6-trimethylbonzoic acid	$C_{10}H_{11}O_4$	195.1 [M - H]$^-$	*P. neglecta* SCSI41403	[4]
6-pentyl-4-methoxy-pyran-2-one		197 [M + H]$^+$;<br>219 [M + Na]$^+$	*P. guepinii*	[60]
Pestalotiopyrone G	$C_{10}H_{12}O_3$	202.9 [M + Na]$^+$	*P. sydowiana*	[61]
Pestalotiopyrone E	$C_{10}H_{14}O_4$	221.0 [M + Na]$^+$	*P. sydowiana*	[61]
LL-P880b	$C_{11}H_{18}O_5$	231.2 [M + H]$^+$	*P. sydowiana*	[61]
Pestalotiopyrone D	$C_{10}H_{14}O_5$	237.0 [M + Na]$^+$	*P. sydowiana*	[61]
Photipyrone	$C_{11}H_{18}O_5$	253.1 [M + Na]$^+$	*P. sydowiana*	[61]
Sesquicaranoic acid B	$C_{15}H_{23}O_4$	267.3 [M - H]$^-$	*P. neglecta* SCSI41403	[4]
Citreorosein	$C_{15}H_9O_6$	285.1 [M - H]$^-$	*P. neglecta* SCSI41403	[4]
Altitoxin B	$C_{15}H_{22}ClO_4$	301 [M - H]$^-$	*P.* sp.	[55]
dehydroisopenicillide	$C_{21}H_{22}O_6$	371.2 [M + H]$^+$	*P. sydowiana*	[61]
3′-O-methyldehydroisopenicillide	$C_{22}H_{24}O_6$	385.4 [M + H]$^+$	*P. sydowiana*	[61]
Pestalotiollide B	$C_{21}H_{22}O_7$	387.4 [M + H]$^+$	*P. sydowiana*	[61]
Pestalotiollide A	$C_{21}H_{22}O_7$	387.4 [M + H]$^+$	*P. sydowiana*	[61]
Monocycloalternarene B	$C_{22}H_{31}O_6$	391.2 [M - H]$^-$	*P. neglecta* SCSI41403	[4]
Pestalone	$C_{21}H_{20}Cl_2O_6$	437.1 [M - H]$^-$;<br>439.1 [M + H]$^+$	*P. neglecta* SCSI41403	[4]
